# Multifaceted roles of extracellular DNA in bacterial physiology

**DOI:** 10.1007/s00294-015-0514-x

**Published:** 2015-09-02

**Authors:** Dina Vorkapic, Katharina Pressler, Stefan Schild

**Affiliations:** Institute of Molecular Biosciences, University of Graz, Humboldtstraße 50, 8010 Graz, Austria

**Keywords:** Neutrophil extracellular traps, Nutrient acquisition, Competence, Nucleoside transporters, Transition fitness, Virulence

## Abstract

In textbooks, DNA is generally defined as the universal storage material for genetic information in all branches of life. Beyond this important intracellular role, DNA can also be present outside of living cells and is an abundant biopolymer in aquatic and terrestrial ecosystems. The origin of extracellular DNA in such ecological niches is diverse: it can be actively secreted or released by prokaryotic and eukaryotic cells by means of autolysis, apoptosis, necrosis, bacterial secretion systems or found in association with extracellular bacterial membrane vesicles. Especially for bacteria, extracellular DNA represents a significant and convenient element that can be enzymatically modulated and utilized for multiple purposes. Herein, we discuss briefly the main origins of extracellular DNA and the most relevant roles for the bacterial physiology, such as biofilm formation, nutrient source, antimicrobial means and horizontal gene transfer.

## Introduction

**Fig. 1:**
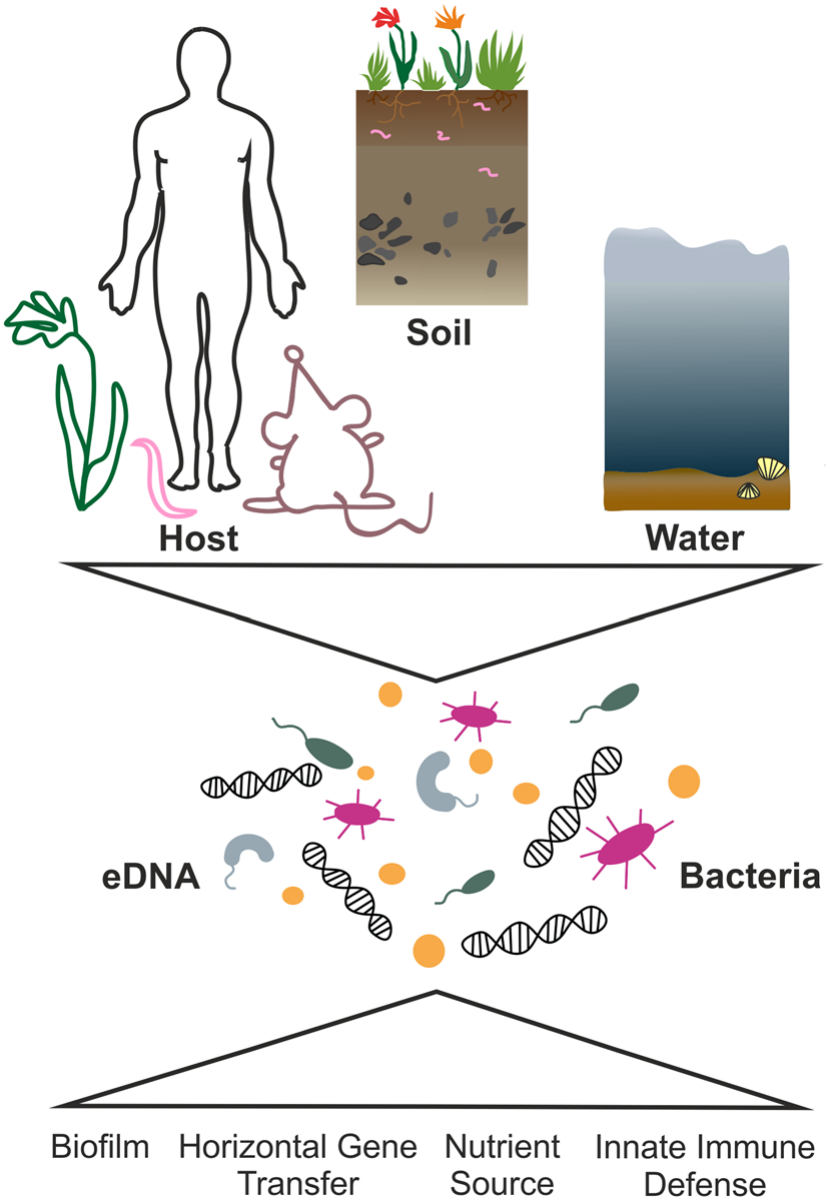
Physiological implications of extracellular DNA (eDNA). Bacteria encounter eDNA, the ubiquitous biopolymer, within the terrestrial and aquatic environments. Pathogenic microorganisms also meet significant amounts of eDNA in the host during infection. Bacteria can utilize eDNA dependent on the environmental condition as nutrient source, for horizontal gene transfer or as biofilm matrix component. By means of degradative enzymes they can not only modulate the polymer, but also evade the innate immune defense mechanism based on eDNA

Extracellular DNA (eDNA) is a ubiquitous biopolymer found in both terrestrial and aquatic ecosystems, reaching concentrations up to 2 μg g^−1^ of the uppermost horizons of soil and up to 0.5 g m^−2^ in the top centimeter of deep-sea sediments (Dell’Anno and Danovaro 2005; Niemeyer and Gessler 2002). The significance of eDNA in bacterial physiology and especially in the life cycle of microbial pathogens drew attention in the past decade, as it became evident that it plays an important role in bacterial pathogenicity, transition fitness, environmental survival and evolution. Bacteria encounter eDNA in the host and outside environment. Back in 1928, F. Griffith reported that pneumococci are capable of transferring genetic information through a process known as transformation, not knowing by then that the transferred material is eDNA (Griffith 1928). In 1956, Catlin observed eDNA as a structural component of bacterial biofilms and in 1980s eDNA was determined as a significant component of small intestinal mucus in rabbit, but its origin in such environments remained speculative (Catlin 1956; Ferencz et al. 1980). Since then, we began to understand how eDNA can be actively secreted or liberated from eukaryotic and prokaryotic cells and how, depending on environmental conditions, bacteria can utilize free DNA as a nutrient source, for recombination into the chromosome, for repair of their own DNA or as a building element in bacterial biofilms (Antonova and Hammer 2015; Dubnau 1999; Flemming and Wingender 2010). In this review, we provide a brief overview of eDNA occurrence and role for bacterial lifestyles, follow the origin of eDNA to its degradation and highlight the importance of eDNA in bacterial life cycle (Fig. 1).

## eDNA is an important component of the biofilm matrix

One stage of bacterial life cycles with relative high local eDNA concentrations are biofilms, which represent multicellular surface communities formed by different microorganisms on biotic or abiotic surfaces. Bacteria can form biofilms in aquatic and terrestrial ecosystems as well as in the host as a survival mode, which protects against harsh environmental conditions such as pH and temperature changes, antimicrobial agents, digestive enzymes, UV light, dehydration or predators (Hall-Stoodley et al. 2004). The substantial segment of extracellular matrix of mature biofilms is produced by the bacteria themselves and consists mainly of proteins, polysaccharides, membrane vesicles and eDNA (Flemming and Wingender 2010). Many studies of biofilms formed by Gram-positive and Gram-negative bacteria such as *Staphylococcus aureus, Listeria monocytogenes, Pseudomonas aeruginosa, Neisseria meningitidis* and *Vibrio cholerae* reveal eDNA to be required for biofilm organization, maturation or even initial attachment of bacteria to the surface (Harmsen et al. 2010; Lappann et al. 2010; Mann et al. 2009; Seper et al. 2011; Whitchurch et al. 2002). Besides its function as a structural component, Gloag and coworkers additionally showed that eDNA was important for coordination of bacterial alignment and movements during biofilm growth in *P. aeruginosa* (Gloag et al. 2013). In contrast, eDNA hinders biofilm development of *Salmonella enterica* ser. Typhimurium and ser. Typhi on abiotic surfaces and prevents *Caulobacter crescentus* swarmer progeny cells from settling into biofilm (Berne et al. 2010; Wang et al. 2014). For certain bacteria that form biofilm inside the host, an additional beneficial feature of eDNA is the induction of genes responsible for resistance to host antimicrobial peptides, as shown for *Salmonella enterica* ser. Typhimurium and *P. aeruginosa* (Johnson et al. 2013; Mulcahy et al. 2008). The presence of eDNA in bacterial biofilms is frequently accompanied by secretion of bacterial nucleases, which makes it a shapeable flexible structural component, adjustable to the needs of the bacterial community. Deletion of extracellular nucleases generally results in compact, thick and unstructured biofilms. Compared to wild-type these mutant biofilms lack visible fluid-filled channels characteristic of mature three-dimensional biofilm matrix (Cho et al. 2015; Kiedrowski et al. 2011; Seper et al. 2011; Steichen et al. 2011). In addition, nucleases are key enzymes, which play a critical role in the degradation of eDNA allowing its utilization as a carbon, nitrogen and phosphate source in nutrient-limited environments (Mulcahy et al. 2010; Pinchuk et al. 2008; Seper et al. 2011). The presence of eDNA in biofilms is not limited to the prokaryotic world. Recently, several reports demonstrate that eDNA contributes also to the maintenance and structural integrity of eukaryotic biofilms such as of *Candida albicans* and *Aspergillus fumigatus,* where it is hypothesized to confer antifungal resistance (Martins et al. 2010; Mathe and Van Dijck 2013; Rajendran et al. 2013). In laboratory research, we tend to see biofilms as single species communities, which is likely not the case in nature. Thus, eDNA could be a vital content produced, modulated and shared for use by multiple species within the biofilm association. Elucidation of such interactions and cross-talks between different species will be a future research task.

## Bacteria encounter eDNA in the host and outside environment

Several mechanisms of eDNA release have been reported in recent years. eDNA can originate from other microorganisms, host or bacteria themselves. For *S. aureus* and *Enterococcus faecalis*, it has been proposed that eDNA release in biofilm development is mainly dependent on two mechanisms of autolysis: programmed cell death (altruistic suicide) and killing of sister cells (fratricide) (Montanaro et al. 2011; Rice et al. 2007; Thomas et al. 2008, 2009). Concordantly, decreased eDNA amounts and reduced biofilm formation have been observed in *N. meningitidis* and *V. choleraeampD* mutants, which exhibit reduced autolysis (Lappann et al. 2010; Seper et al. 2011). In dual species cultures, *Streptococcus sanguinis* and *Streptococcus gordonii* release eDNA in a process induced by pyruvate oxidase-dependent production of H_2_O_2_. Such an autolysis-independent DNA release is suggested to be an adaptation to the competitive oral biofilm environment, where both species can efficiently compete with other H_2_O_2_-sensitive colonizers and autolysis could create open spaces for competitors to invade (Kreth et al. 2009). Besides bacterial eDNA release, eukaryotes can also be a donor of eDNA. For example, throughout the bacterial disease, several pathogens secrete pore-forming toxins, e.g., the alpha-hemolysin of *E. coli*, the cytolysin of *V. cholerae*, the listeriolysin O of *L. monocytogenes*, the alpha-toxin of *S. aureus*, or toxins that act as inhibitors on the protein synthesis, e.g., exotoxin A of *P. aeruginosa*, to induce apoptosis, necrosis and lysis of host cells and therefore promote liberation of DNA (Bayles et al. 1998; Fernandez-Prada et al. 1998; Guzman et al. 1996; Jonas et al. 1994; Merrick et al. 1997; Morimoto and Bonavida 1992; Moss et al. 1999; Rogers et al. 1996; Russo et al. 2005; Saka et al. 2008). Notably, in a variety of bacteria such as *Streptococcus pneumoniae*, *S. aureus* and *N. meningitidis*, typical cytoplasmic proteins are found to be be released via non-classical signal-dependent pathways (Bergmann et al. 2001; Gotz et al. 2015; Kolberg et al. 2008). Originally thought to occur via cell lysis, there is mounting evidence that excretion of such proteins involves a programmed process as part of their normal cell cycle, which could also be a relevant mechanism for eDNA liberation (Ebner et al. 2015a, b). Another source of eDNA in the host is a defense mechanism by the innate immune system known as neutrophil extracellular traps (NETs). NETs originate from neutrophils undergoing a programmed cell death upon activation through a variety of microbial pathogens (Fuchs et al. 2007). They release nuclear or mitochondrial DNA backbone associated with histones and granular and cytoplasmic proteins to capture and kill the intruders (Brinkmann et al. 2004). The immobilization furthermore prevents spread of the microbes from the initial site of infection and recruits additional professional phagocytes to eliminate the pathogens. In return, the microbes have evolved to escape these disarming and killing traps. The best strategy to evade NETs is to actively degrade them. Since the main component is DNA, NETs can be efficiently degraded by DNases, which has been demonstrated to be relevant for virulence fitness of several bacteria, including the group A *Streptococcus*, *S.aureus*, *S.pneumoniae* and *V.cholerae* (Beiter et al. 2006; Berends et al. 2010; Brinkmann et al. 2004; Buchanan et al. 2006; Seper et al. 2013). Recently, it was shown that *S.aureus* can further convert the DNA derived from NETs to 2′-deoxyadenosine by the activity of an adenosine synthase A on top of the endo-exonuclease Nuc (Thammavongsa et al. 2011, 2013). The released 2′-deoxyadenosine triggers apoptosis of macrophages via accumulation of intracellular dATP and activation of caspase-3 (Koopman et al. 1994; Thammavongsa et al. 2013). Thus, *S.aureus* not only evades NETs, but also turns the DNA of this defense mechanism back against the host by the use of bacterial enzymes.

Additionally to their function of releasing pathogens from NETs, nucleases can also mediate dispersal of biofilms. For example the two extracellular nucleases of *V. cholerae* are crucial for biofilm detachment (Seper et al. 2011). The impact of this biofilm dispersion is highlighted by the in vivo fitness of *V. cholerae*, the causative agent of the waterborne diarrheal disease cholera. *V. cholerae* biofilm clumps derived from the aquatic reservoir are a likely form in which the pathogen is taken up by humans. Such infectious aggregates provide a concentrated bacterial dose and are protected against acids or bile salts (Hall-Stoodley and Stoodley 2005; Hartley et al. 2006; Huq et al. 1996; Nalin et al. 1978, 1979; Pruzzo et al. 2008; Zhu and Mekalanos 2003). Indeed, biofilm-derived *V. cholerae* outcompete their planktonic counterparts in the murine model (Tamayo et al. 2010). However, for successful colonization in the small intestine *V. cholerae* has to detach from the biofilm to adhere and penetrate through the mucosal layer aided by motility, which requires a planktonic state (Butler and Camilli 2005; Freter and Jones 1976; Freter and O’Brien 1981; Zhu and Mekalanos 2003). Concordantly, biofilm clumps of extracellular nuclease mutants are attenuated in vivo, as biofilm detachment of these mutants is massively decreased (Seper et al. 2011).

## eDNA serves as element for evolution and nutrient source

Extracellular DNA is also a pool for horizontal gene transfer (HGT), which is defined by the utilization of exogenous DNA for the purpose of genetic recombination and requires natural competence of bacterial cells to yield evolutionarily favorable properties. Generally, active DNA release for the purpose of HGT via bacterial conjugation occurs via type IV secretion system, which requires cell–cell contact. To our best knowledge, secretion of DNA via type IV secretion system without requirement of a physical cell–cell contact has only been documented for *Neisseria gonorrhoeae*. The consequence of such system is spreading genetic information through the population and the possibility of using eDNA for nutrient acquisition or biofilm formation without reducing cell population and promoting host immune response (Hamilton et al. 2005; Zweig et al. 2014). eDNA can also be found in association with outer membrane vesicles (OMVs) of Gram-negative species (Dorward et al. 1989; Garon et al. 1989; Loeb et al. 1981). Interestingly, OMVs in *Helicobacter pylori* and *Pseudomonas putida* promote biofilm formation (Baumgarten et al. 2012; Yonezawa et al. 2009). The same is known for *Acinetobacter baumannii* where release of OMVs is one of the main mechanisms that contribute to total availability of eDNA (Sahu et al. 2012). The export of DNA via membrane vesicles (MVs) has been observed for a long time as a characteristic of Gram-negative species (Dorward and Garon 1990); however, a recent study by Liao et al. showed the presence of eDNA in MVs of the Gram-positive bacterium *Streptococcus mutans* (Liao et al. 2014). MVs containing DNA increase the efficiency of DNA uptake and genetic recombination, as it has been shown for example in *H. influenzae* and *E. coli*, most likely because DNA in vesicles is protected from degradation and vesicles may efficiently fuse back into the cell membrane (Deich and Hoyer 1982; Kahn et al. 1983; Renelli et al. 2004; Yaron et al. 2000). Thus, MVs might act as DNA delivery vehicles, but the exact localization of the DNA, the molecular mechanism of DNA deposition in vesicles and later uptake in the donor cell as well as the relevance of MVs-mediated HGT need to be investigated in the future. A recent work of Borgeaud et al. demonstrates that *V. cholerae* is capable of type VI secretion system-mediated killing of nonimmune neighboring cells and liberation of their DNA, which can subsequently act as eDNA for HGT (Borgeaud et al. 2015).

Other examples for HGT include *Campylobacter jejuni* where eDNA facilitates transfer of genetic traits between bacteria in biofilm, which can contribute to spread of antimicrobial resistance (Brown et al. 2015). Furthermore, antibiotic resistances encoded on plasmids can spread via transformation in multispecies oral biofilms (Hannan et al. 2010). Notably, regulatory circuits of biofilm formation, quorum sensing (QS), carbon catabolite repression (CCR) and competence are frequently linked in bacteria (Spoering and Gilmore 2006; Yang and Lan 2015). In *S. mutans*, QS signal stimulates the uptake of eDNA causing cells in biofilm to undergo an enhanced competence induction (Håvarstein and Morrison 1999; Li et al. 2001). Moreover, transcription factor CcpA regulates competence and biofilm development in *S. gordonii* during CCR to ensure that cell energy is used for uptake of preferable carbon source (Zheng et al. 2012). In *H. influenzae,* competence is regulated by the availability of nucleic acid precursors, which is under control of CRP-dependent regulon (MacFadyen et al. 2001; Redfield et al. 2005). As nutrient starvation is the main signal for competence induction in *H. influenzae*, it has been suggested that it emerged as a ‘DNA for food’ uptake system, rather than being used for HGT (Redfield 1993). In addition, the competence system in *E.coli* possibly favors utilization of DNA for the purpose of nutrient acquisition rather than processing it for genetic transformation (Finkel and Kolter 2001).

One of the intensively studied regulatory systems of competence is *V. cholerae*. Meibom et al. showed that *V. cholerae* induces natural competence when growing on chitin, an abundant biopolymer that can be readily used as a carbon source, suggesting another example of bacterial competence during CCR (Meibom et al. 2005). Thus, it is not surprising that the above-mentioned type VI secretion system in *V. cholerae*, which promotes bacterial predation when growing on chitin, is a part of the competence regulon (Borgeaud et al. 2015). Chitin utilization and competence genes in *V. cholerae* are under positive control of the QS regulator HapR, the cytidine repressor CytR and CRP, a global regulator of CCR (Antonova et al. 2012; Antonova and Hammer 2015). Interestingly, CytR acts on the competence genes as an anti-activator in concert with the CRP–cAMP complex, while free cytidine is a repressor for natural competence (Antonova et al. 2012). In contrast, HapR acts as a repressor for the secreted endonuclease Dns, and CytR negatively controls nucleoside uptake via inner membrane transporters in *V. cholerae* (Blokesch and Schoolnik 2008; Gumpenberger et al. 2015; Haugo and Watnick 2002; Lo Scrudato and Blokesch 2012). Additionally, the CRP–cAMP complex positively regulates the nucleoside uptake (Gumpenberger et al. 2015). Taken together, absence of PTS sugars resulting in high levels of cAMP is a prerequisite for activation of competence and utilization of DNA as nutrient source. At low cell densities and presence of nucleotide expression of genes involved in utilization of eDNA, including the secreted endonuclease Dns may facilitate survival using eDNA as nutrient source. At high cell density and presence of nutrient sources other than nucleotides, eDNA utilization is repressed and the uptake of intact DNA and potential genome diversification by HGT is in favor.

The complex pathway of eDNA degradation in *V. cholerae* and the subsequent utilization of nucleotides as phosphate, carbon and nitrogen source has recently been solved. Extracellular nucleases Xds and Dns are both induced under low phosphate conditions (McDonough et al. 2014; Seper et al. 2011), causing extracellular accumulation of nucleotides. Nucleotides can transit through outer membrane via pore-forming outer membrane protein OmpK, a homolog of Tsx in *E. coli* (Maier et al. 1988; Osborn and Wu 1980), and are subsequently dephosphorylated in the periplasm via three periplasmic phosphatases (nucleotidases) with different specificities. In detail, UshA facilitates phosphate removal from all four 5′ deoxynucleotides, CpdB from 3′AMP, 3′dGMP and 3′TMP and PhoX preferentially from 3′dAMP and 3′dCMP (McDonough et al. 2015). Free nucleosides are then readily taken up in the cell by three NupC nucleoside transport systems and used as a source of carbon and nitrogen (Gumpenberger et al. 2015), while phosphate is taken up by the Pst/PhoU system which has been identified in the genome and demonstrated as active (Heidelberg et al. 2000; McDonough et al. 2014; Pratt et al. 2009). All three nucleoside transport systems are functional, but exhibit slightly different nucleobase specificity and activities (Gumpenberger et al. 2015). Interestingly, a mutant lacking all three nucleoside transporters shows no attenuation in vivo, but exhibits a fitness disadvantage when transitioning from the host to nutrient-poor aquatic environment (Gumpenberger et al. 2015). Similar observations have been previously reported for hexose-6-phosphate uptake in *V. cholerae* (Moisi et al. 2013). Notably, *V. cholerae* is capable of storing carbon and phosphate in the form of glycogen or polyphosphate, respectively (Bourassa and Camilli 2009; Jahid et al. 2006). Such findings reinforce the hypothesis in which nucleoside uptake genes, like many other genes induced in later stages of the *V. cholerae* infection, do not play a direct role in the in vivo fitness, but rather increase the transition fitness of the pathogen due to the severe drop in nutrient source availability upon exiting from the host into the aquatic environments (Schild et al. 2007).

Originally described in *E. coli*, NupC transport system was shown to be a member of concentrative nucleoside transporter (CNT) family, driven by H^+^-motive force and discriminative for adenosine and cytidine (Munch-Petersen and Mygind 1976; Patching et al. 2005). Several homologs of *E. coli* NupC have been known to act as nucleoside transporters in *S. aureus*, *H. pylori* or *Bacillus subtilis* where nucleosides can be used as energy source or for de novo synthesis of nucleotides (Kriegeskorte et al. 2014; Miller et al. 2012; Saxild et al. 1996). Interestingly, three NupC systems of *V. cholerae* are the first bacterial nucleoside transport systems, which use Na^+^ for effective transport like their homologs hCNT or rCNT in humans or rodents, respectively (Johnson et al. 2012; Ritzel et al. 1997). Therefore*, V. cholerae* might be an ideal bacterial candidate for investigating the cellular uptake route for many cytotoxic nucleoside derivatives used in the treatment of viral and neoplastic diseases (Baldwin et al. 1999; Johnson et al. 2012, 2014).

## Conclusion and future perspectives

Throughout the evolution, bacteria have been forced to acquire mechanisms, which would enable fast regulation of gene expression in response to different environmental signals. Such genes are often involved in major physiological changes, such as transition from host to the outside environment and switch from planktonic to sessile state or vice versa. Especially, biofilm formation is a survival strategy of many bacteria and can be seen in the host, or aquatic or terrestrial habitat. Cells in the biofilm are embedded in the dynamic matrix where they reach homeostasis and are organized to exploit all available nutrients (Sutherland 2001). Particularly, eDNA has recently emerged as an important component of the biofilm matrix which forms agglomerates with other matrix components and therefore acts as a ‘glue’ between cells, contributing to its stability (Peterson et al. 2013). Thus, eDNA can be seen as a potential target for biofilm control, as destabilizing of eDNA interactions with other matrix components generally leads to destabilization of biofilm and increased antibiotic susceptibility (Okshevsky et al. 2015). In this review, we also focused on degradation of eDNA and its subsequent uptake into the cell via nucleoside transporters in human pathogen *V. cholerae,* which like many bacteria can utilize eDNA as source of nutrients. Yet, many questions are left unanswered—Why are *V. cholerae* transporters sodium dependent? Why *V. cholerae* needs three transporters? The answer could lay in the observation of its life cycle, which is marked by distinct changes in nutrient availability, osmolarity, pH and temperature. Similarly, *B. subtilis* also encodes three nucleoside transporters. *B. subtilis* enters a dormant stage (spore) when nutrients in the environment are deprived. The existence of three nucleoside transport systems may enable bacteria to selectively take up compounds relevant for a specific stage of the life cycle. Concordantly, the regulation of such systems ensures that, once the cell has started the differentiation or adaptation, they can be completed even with environmental changes (Beaman et al. 1983). Whether this is also true for other pathogens with complex life cycles, which are able to make use of eDNA as a nutrient source, remains to be elucidated.

## Abbreviations

eDNA: Extracellular DNA
NETs: Neutrophil extracellular traps
HGT: Horizontal gene transfer
QS: Quorum sensing
MVs: Membrane vesicles
OMVs: Outer membrane vesicles
CCR: Carbon catabolite repression
